# Ru/Ir‐Based Electrocatalysts for Oxygen Evolution Reaction in Acidic Conditions: From Mechanisms, Optimizations to Challenges

**DOI:** 10.1002/advs.202309364

**Published:** 2024-03-19

**Authors:** Rong Qin, Guanzhen Chen, Xueting Feng, Jiena Weng, Yunhu Han

**Affiliations:** ^1^ Institute of Flexible Electronics (IFE) Ningbo Institute of Northwestern Polytechnical University Frontiers Science Center for Flexible Electronics Northwestern Polytechnical University Xi'an Shaanxi 710129 China

**Keywords:** acidic electrolyte, opportunities and challenges, oxygen evolution reaction activity and stability, Proton exchange membrane water electrolysis, Ru/Ir‐based electrocatalysts

## Abstract

The generation of green hydrogen by water splitting is identified as a key strategic energy technology, and proton exchange membrane water electrolysis (PEMWE) is one of the desirable technologies for converting renewable energy sources into hydrogen. However, the harsh anode environment of PEMWE and the oxygen evolution reaction (OER) involving four‐electron transfer result in a large overpotential, which limits the overall efficiency of hydrogen production, and thus efficient electrocatalysts are needed to overcome the high overpotential and slow kinetic process. In recent years, noble metal‐based electrocatalysts (e.g., Ru/Ir‐based metal/oxide electrocatalysts) have received much attention due to their unique catalytic properties, and have already become the dominant electrocatalysts for the acidic OER process and are applied in commercial PEMWE devices. However, these noble metal‐based electrocatalysts still face the thorny problem of conflicting performance and cost. In this review, first, noble metal Ru/Ir‐based OER electrocatalysts are briefly classified according to their forms of existence, and the OER catalytic mechanisms are outlined. Then, the focus is on summarizing the improvement strategies of Ru/Ir‐based OER electrocatalysts with respect to their activity and stability over recent years. Finally, the challenges and development prospects of noble metal‐based OER electrocatalysts are discussed.

## Graphical abstract

In order to increase the efficiency of hydrogen production by water splitting, it is aimed to improve the anode oxygen evolution reaction (OER). The Ru/Ir‐based OER electrocatalysts are briefly categorized according to their existence forms and outline the OER catalytic mechanism. Importantly, strategies for the improvement of Ru/Ir‐based OER electrocatalysts in terms of activity and stability in recent years are summarized.

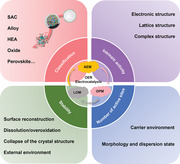

## 1 Introduction

With the rapid development of human society, the consumption of fossil fuels has caused many serious problems such as energy crises, global climate change, and environmental pollution, etc.^[^ ^1^, ^2^, ^3^ ^]^ Developing efficient and stable energy conversion and storage technologies to replace traditional fossil fuel resources is an effective strategy for addressing these issues.^[^ ^4^ ^]^ In recent years, the renewable energy sources such as solar and wind power have evolved considerably.^[^ ^5^, ^6^, ^7^, ^8^, ^9^ ^]^ However, these renewable energy sources are susceptible to environmental factors such as time, season, weather, and location, resulting in unstable and irregular output.^[^ ^10^, ^11^ ^]^

**Scheme 1:**
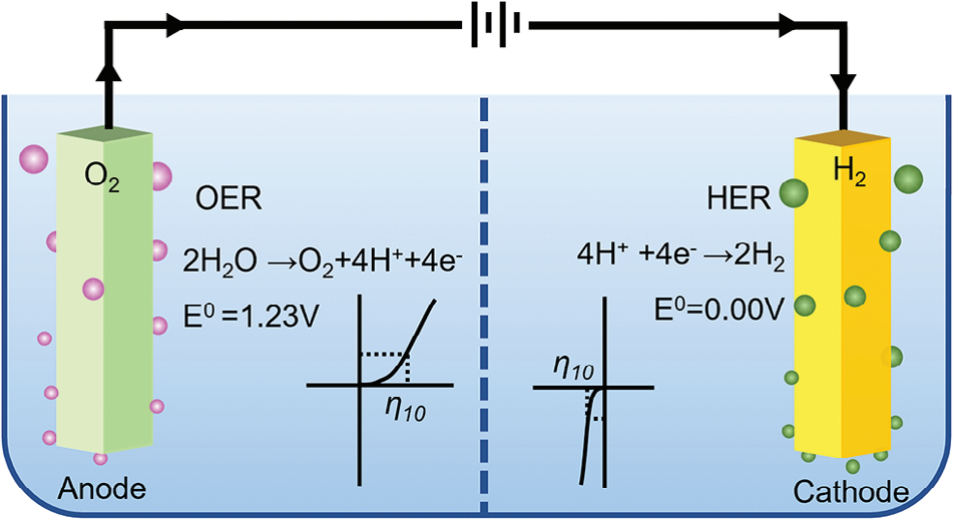
Electrochemical water splitting in acidic condition.

The utilization of renewable energy generation for the water splitting to produce green hydrogen has been identified as a key sustainable energy technology strategy for decarbonization in the industrial and transportation sectors.^[^ ^12^, ^13^, ^14^ ^]^ Numerous electrolysis cell devices can convert intermittent renewable energy sources such as solar, wind and tidal energy into H_2_ and O_2_ using water splitting technology, which can be reconverted to electricity in a fuel cell when needed.^[^ ^15^, ^16^ ^]^ The technology is environmentally friendly, utilizes abundant raw materials, and is crucial for the development of hydrogen energy.^[^ ^15^, ^16^, ^17^, ^18^, ^19^ ^]^ Water splitting consists of two half‐reactions, the hydrogen evolution reaction (HER) and the oxygen evolution reaction (OER). The OER occurs at the anode, while the HER is coupled with the OER to form H_2_ at the cathode (**Scheme** **1**).^[^ ^20^ ^]^ Among various water splitting technologies, proton exchange membrane water electrolysis (PEMWE) has many advantages such as high hydrogen purity, high efficiency, and excellent high‐pressure tolerance.^[^ ^21^ ^]^ In addition, it also has the advantages of rapid response, high energy conversion efficiency, low operating cost, easy stacking and scaling.^[^ ^22^, ^23^, ^24^, ^25^ ^]^ Therefore, PEMWE is considered as one of the ideal technologies for converting renewable energy into hydrogen. The anodic oxidation environment of the PEMWE has a low pH, high oxygen concentration, high potential, and the OER process involves four‐electron transfer, resulting in a much larger overpotential for the OER process than for the HER process.^[^ ^26^, ^27^ ^]^ Therefore, the kinetics of OER determines the overall hydrogen production efficiency, and efficient electrocatalysts are needed to overcome the high overpotential and sluggish kinetics of OER.^[^ ^28^, ^29^ ^]^

The “volcano diagram” calculated by density functional theory (DFT) predicts that noble metals generally have suitable free energies for the adsorption of intermediates and therefore have better OER activity.^[^ ^30^ ^]^ Compared with non‐noble metal electrocatalysts, noble metal electrocatalysts have higher activity, more selectivity and better stability.^[^ ^31^ ^]^ First, the *d*‐electron orbitals of the noble metal electrocatalysts Ru and Ir are unfilled, and the reactants are easily adsorbed on the surface of the electrocatalysts with a small energy level spacing, and the adsorption strengths of the reactants and intermediates are moderate, which is favorable for the formation of intermediate reactive species and the catalytic activity is high. Second, noble metal electrocatalysts can coordinate with a variety of substances in various forms and have strong selectivity in catalytic reactions, so they are widely used in the field of catalysis.^[^ ^32^, ^33^, ^34^ ^]^ In addition, non‐noble metal electrocatalysts have made great progress in terms of activity and stability, but most of them are applicable to alkaline conditions, while noble metal electrocatalysts are generally chemically stable and can adapt to more demanding catalytic environments.^[^ ^7^, ^35^, ^36^, ^37^, ^38^ ^]^ The development of the water splitting industry requires electrocatalysts with superior activity and stability over a wide pH range, longer service life, and higher catalyst utilization. Based on the above analysis, noble metal electrocatalysts have excellent OER intrinsic catalytic activity, and noble metal‐loaded OER electrocatalysts are expected to be the main cost driver for achieving megawatt scale and above.^[^ ^39^ ^]^

Despite the great efforts made by researchers in developing OER electrocatalysts for acidic electrolyte, the anode OER electrocatalysts that have been used in commercial PEMWE are still dominated by Ru/Ir‐based electrocatalysts. IrO_2_ and RuO_2_ are the most widely used OER catalysts in practical applications, and IrO_2_ is the only catalyst with sufficient OER activity and durability for use in practical PEMWE environments.^[^ ^40^, ^41^ ^]^ As the rarest element in the world, Ir can only be produced in 6–8 tons per year, which is far from being able to satisfy the large‐scale use in PEMWE.^[^ ^42^ ^]^ Ru has advantages in terms of reserves and costs, Ru‐based electrocatalysts are considered to be the most advanced OER electrocatalysts under acidic conditions except Ir‐based electrocatalysts, but the long‐term stability of Ru‐based electrocatalysts under acidic conditions is far lower than that of Ir‐based electrocatalysts, even though the activity of Ru‐based electrocatalysts is slightly higher than that of Ir‐based electrocatalysts, which would seriously restrict their large‐scale use in PEMWE.^[^ ^43^, ^44^, ^45^ ^]^ To solve the mentioned difficulties faced by PEMWE, a lot of efforts from researchers are needed to further develop new technologies and methods to balance the relationship between the activity and stability of Ru‐based OER catalytic materials.^[^ ^28^, ^46^ ^]^

**Scheme 2:**
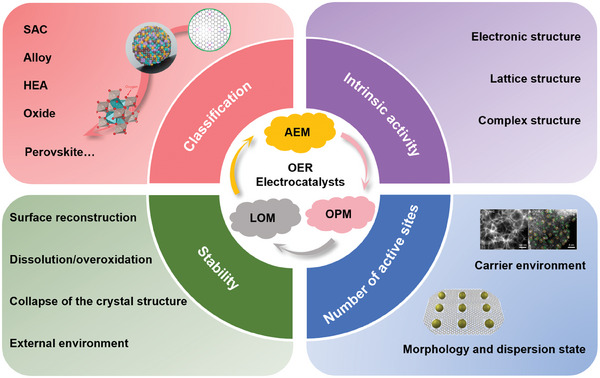
Schematic of the main content for acidic Ru/Ir‐based OER electrocatalysts in this review, including OER mechanism, classification of electrocatalysts, intrinsic activity and the number of active sites of the electrocatalysts, and the strategy for stability enhancement. (Single‐atom. Reproduced with permission.^[^ ^47^ ^]^ Copyright 2020, American Chemical Society. High‐entropy alloys. Reproduced with permission.^[^ ^48^ ^]^ Copyright 2022, Springer Nature. Metal oxides. Reproduced with permission.^[^ ^49^ ^]^ Copyright 2022, Elsevier. Carrier environment. Reproduced with permission.^[^ ^50^ ^]^ Copyright 2023, American Chemical Society. Morphology and dispersion state. Reproduced with permission.^[^ ^51^ ^]^ Copyright 2021, The American Association for the Advancement of Science.

In this review, an overview of recent advances in noble metal Ru/Ir‐based OER electrocatalysts is highlighted. First, the catalytic mechanism of OER process is briefly introduced. Subsequently, the electrocatalysts are briefly categorized according to the existence forms of Ru/Ir species, such as single‐atom, alloy and metal oxides. Then, the review focuses on summarizing and presenting the strategies to improve the activity and stability of Ru/Ir‐based OER electrocatalysts. Finally, the challenges and development prospects of Ru/Ir‐based OER electrocatalysts are discussed to provide guidance for the design and synthesis of highly active and stable noble metal Ru/Ir‐based OER electrocatalysts (**Scheme** **2**).

## 2 The Mechanism of OER

**Scheme 3:**
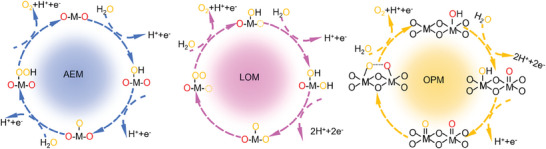
Schematic illustration of the adsorption evolution mechanism (AEM), lattice oxygen‐evolution mechanism (LOM) and oxide path mechanism (OPM) in the OER process under acidic conditions. (Oxygen plotted in dashed lines represents oxygen vacancy and M stands for Ru/Ir).

During the process of water splitting, the primary objective of the electrocatalysts is to lower the energy barrier of OER, promote the evolution and release of oxygen, and enhance the reaction rate.^[^ ^52^ ^]^ The catalyst undergoes redox cycling and the valence state of the active center is restored from the high valence state of the OER process to the initial active state. As depicted in **Scheme** **3**, there are three commonly used models, namely, adsorption evolution mechanism (AEM), lattice oxygen‐evolution mechanism (LOM) and oxide path mechanism (OPM) for describing the OER mechanism.^[^ ^53^, ^54^, ^55^, ^56^ ^]^

### 2.1 Adsorption Evolution Mechanism (AEM)

Taking acidic conditions as an example, the AEM first involves the adsorption of H_2_O on the active surface sites of the electrocatalyst, and the release of H^+^ and an electron at a electrocatalytic site to form an M‐OH intermediate (HO*) . Subsequently, one H^+^ and one electron are released from the HO* to form an O*. Then, another H_2_O nucleophilic attacks the O* to generate an HOO*, releasing both an H^+^ and an electron. Finally, within the HOO*, three electrons from the OOH anion are transferred to the M cation, regenerating the active site and releasing O_2_. At the same time, one H^+^ and one electron are also released.^[^ ^54^ ^]^ The binding energies of both HO* and HOO* can be used as rate‐determining steps (RDS), and the difference in binding energies between HO* and HOO* can be used as a descriptor for OER activity, a theoretically considerable overpotential of 370 mV is observed in AEM, suggesting that it is difficult for AEM‐dominated electrocatalysts to exceed the theoretical overpotential to enhance activity.^[^ ^57^ ^]^ However, the OER onset overpotentials of many reported advanced electrocatalysts are much lower than the minimum value proposed by AEM, suggesting that a dynamic structural evolution of the catalyst surface occurs during the reaction process. Therefore, a LOM with triggered lattice oxygen is proposed to address the surface dynamic evolution problem and to account for the intrinsic overpotential hindrance in AEM.^[^ ^58^ ^]^

### 2.2 Lattice Oxygen‐Evolution Mechanism (LOM)

Since the activity of some electrocatalysts exceeds the limit described by the volcano relationship curve, a lattice oxygen‐based LOM mechanism has been proposed. In the LOM pathway, the formation process involving O* and HO* are similar to that of AEM. Under the action of oxidation potential, one of the two lattice oxygen atoms connected to the metal site moves to the other oxygen atom, forming O─O bonds directly, and the lattice oxygen participates in the reversible generation of O_2_ through surface oxygen vacancies. The HOO* are not formed in the LOM process, and the overpotential limitation in AEM can be effectively reduced.^[^ ^59^ ^]^ In the O_2_ generated by AEM, all oxygen atoms come from the adsorbent H_2_O. After oxygen atoms are adsorbed on the catalyst surface, electron transfer and conformational rearrangement occur, forming active sites on the catalyst surface. The AEM model mainly focuses on the adsorption situation on the catalyst surface and its interaction with the surrounding environment. In LOM, all or part of the oxygen atoms in the generated O_2_ come from the lattice oxygen in the catalyst. The interaction between lattice oxygen sites and active sites on the catalyst surface forms the active site of the catalyst. The LOM model primarily focuses on the position, activity, and potential dependence of the lattice oxygen site. LOM generally produces higher OER activity than AEM, but the oxygen vacancies generated in LOM may not be filled by oxygen atoms in subsequent catalytic cycles, resulting in severe metal dissolution and structural instability.^[^ ^60^, ^61^, ^62^ ^]^

### 2.3 Oxide Path Mechanism (OPM)

In addition to the AEM and LOM theories, researchers have proposed OPM to explain the activity and stability of OER electrocatalysts beyond theoretical values. In OPM, there is no need for lattice oxygen to be involved and the active sites can act synergistically to produce only O* and HO*, without oxygen vacancies as well as HOO* generation, and to allow the triggering of direct coupling of O* radicals to produce O_2_. Therefore, the OPM pathway requires more stringent geometrical. Therefore, the OPM pathway has more stringent requirements for the geometric configuration of metal active sites.^[^ ^55^ ^]^

These three models provide different perspectives for explaining the activity of OER electrocatalysts. The mechanism of OER electrocatalysts is a complex process involving the structure of the catalyst surface, active sites, electron transfer, and the generation of oxide species. AEM is described by the intermediate adsorption energy as a descriptor, to achieve optimal OER activity, the binding strength of the reaction intermediate to the active site should be kept moderate, which is favorable for the adsorption/desorption to reach an equilibrium. LOM can break the overpotential constraints of AEM by introducing defects (e.g., oxygen vacancies, etc.) to provide more active OER activity, and in the case of RuO_2_ and IrO_2_ with perfect (110) and (211) surfaces, AEM is more favorable than LOM. The introduction of defects tends to shift from AEM to LOM, but in the LOM‐guided OER process, due to the continuous formation of oxygen vacancies and the dissolution of M, it leads to problems such as surface structural remodeling and oxygen diffusion, which is not conducive to the durability of the electrocatalysts.^[^ ^63^, ^64^ ^]^ The OPM ensures the activity of the electrocatalysts as well as their stability.^[^ ^65^ ^]^ When interpreting and designing OER catalysts, satisfactory electrocatalysts can be achieved through rational conversion and regulation of different mechanisms. Considering both the AEM, LOM and OPM models allows for a more comprehensive understanding of the activity mechanism of OER electrocatalysts and provides guidance for the further design and development of efficient and stable electrocatalysts. Current research primarily focuses on exploring active sites, regulating catalyst surface structures, optimizing electron transfer processes, and other aspects to improve the performance and stability of OER electrocatalysts. The mechanism of OER electrocatalysts remains a hot research field, with many challenges and unresolved issues at present.

## 3 Classification of Noble Ru/Ir‐Based OER Electrocatalysts

**Figure 1:**
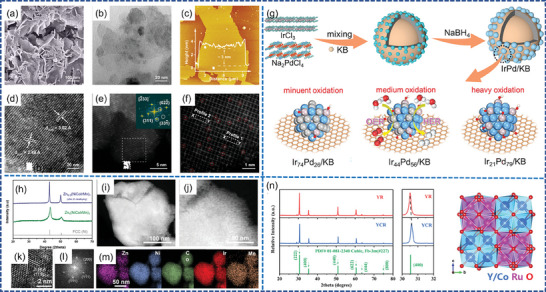
a) Scanning electron microscope (SEM), b) Transmission electron microscope (TEM), c) Atomic force microscope (AFM) and d) High resolution transmission electron microscope (HR‐TEM) images of Ir‐Co_3_O_4_. e) Aberration‐corrected high‐angle‐annular‐dark‐field scanning transmission electron microscopy (AC‐HAADF‐STEM) image and the corresponding fast Fourier transform (FFT) image of Ir‐Co_3_O_4_. f) The enlarged area in (e), with the Ir single atoms marked in circles. Reproduced with permission.^[^ ^72^ ^]^ Copyright 2022, Springer Nature. g) Illustration image for the synthetic process and water splitting mechanism of alloy IrPd/KB. Reproduced with permission.^[^ ^79^ ^]^ Copyright 2023, John Wiley and Sons. h) X‐ray diffraction (XRD) of ZnNiCoIrMn after Zn dealloying. i,j) Scanning transmission electron microscopy (STEM), k) HR‐TEM image, l) Selective area electron diffraction (SAED) pattern, and m) Energy dispersive X‐ray spectroscopy (EDX) elemental mapping of ZnNiCoIrMn. Reproduced with permission.^[^ ^83^ ^]^ Copyright 2023, John Wiley and Sons. n) Powder X‐Ray diffraction (PXRD) patterns of Y_2_Ru_2_O_7_ (YR), Y_1.75_Co_0.25_Ru_2_O_7_ (YCR) and Crystal structure of cubic Y_2‐x_Co_x_Ru_2_O_7_. Reproduced with permission.^[^ ^91^ ^]^ Copyright 2023, John Wiley and Sons.

Noble metal Ru/Ir‐based materials are the most dominant acidic OER electrocatalysts at present, and can be classified into three categories based on the presence of Ru and Ir species in their forms: Ru/Ir single‐atom catalysts (SACs), Ru/Ir alloy catalysts, and Ru/Ir metal oxides catalysts. Researchers have made great efforts to improve the OER activity and long‐term stability of the three types of OER electrocatalysts (**Figure** **1**).

### 3.1 Single Atom

The reduction of noble metal content in electrocatalysts while maintaining the activity and stability of the electrocatalysts is the first fundamental for the development of advanced noble metal‐based OER electrocatalysts. SACs have been widely focused on and employed in OER by researchers due to their maximized atom utilization, high activity and stability.^[^ ^66^, ^67^, ^68^ ^]^ Because the coordination environment of the metal active site of SACs is precisely controllable, the electronic structure of the metal site can be changed by adjusting the coordination, thus modulating the electron density of the metal active site, the interaction with the reactants, and thus affecting the adsorption strength of the reaction intermediates, and greatly improving the catalytic performance.^[^ ^69^, ^70^, ^71^ ^]^ Zhu et al. prepared Ir‐Co_3_O_4_ catalyst by doping dispersed Ir atoms into spinel Co_3_O_4_. Ir single atoms increased electronic conductivity and decreased the adsorption energy barrier. Ir‐Co_3_O_4_ exhibited an overpotential of 236 mV and long‐term stability for nearly 30 h at 10 mA cm^−2^ in acidic media (Figure 1a–f).^[^ ^72^ ^]^ Zhao et al. incorporated Ir single atoms into the lattice structure of transition metal oxides. The obtained Ir_0.08_Co_2.92_O_4_ NWs with low Ir content exhibited excellent acidic OER performance with an overpotential of only 189.5 mV, maintaining excellent durability for over 100 h at 10 mA cm^−2^.^[^ ^73^ ^]^ Compared with Ir, Ru is much more abundant than Ir and has an obvious cost advantage. Rong et al. prepared Ru/Co‐N‐C by anchoring atomically dispersed Ru/Co double sites on N‐doped carbon, and the electronic structure of Ru could be effectively changed by introducing Co‐N_4_ sites, and the overpotential of the OER of the Ru/Co‐N‐C catalyst was 232 mV at a current density of 10 mA cm^−2^ and maintained over 20 h.^[^ ^74^ ^]^ To improve the stability and activity of single‐atom electrocatalysis, single atoms were anchored back to large substrates by strong metal‐carrier interactions. Zhang et al. prepared single‐atom catalysts (h‐HL‐Ir SACs) by confining the active Ir atoms to amino‐functionalized carbon substrates (NH_2_C) and introducing a large number of *d*‐band holes, the rapid accumulation of HOO* and HO* on the cavity‐modulated Ir sites allowed for fast OER kinetics. As a result, this well‐designed h‐HL‐Ir SACs showed excellent performance in acidic OER, with an overpotential of 216 mV at 10 mA cm^−2^, a Tafel slope of 43 mV dec^−1^, and no significant attenuation of catalytic activity after 60 h of operation in an acidic environment.^[^ ^75^ ^]^

### 3.2 Alloy

Although the Ru/Ir‐based single‐atom OER electrocatalysts exhibit excellent mass activity, the stability of Ru/Ir‐based single‐atom OER electrocatalysts in acidic electrolyte is still slightly insufficient due to the susceptibility of the Ru/Ir active sites to migratory agglomeration, delocalization, and dissolution of the carriers during the OER process.^[^ ^15^, ^63^, ^76^ ^]^ Alloying of noble metals is one of the strategies to reduce the consumption of noble metals and still maintain the initial activity of noble metals.^[^ ^77^ ^]^ Moreover, when the active noble metal is alloyed with other metals or non‐metals, electron transfer between the other metals/non‐metals and the active noble metal can further optimize the catalytic activity of the noble metal.^[^ ^78^ ^]^ Yang et al. prepared IrPd alloy electrocatalysts supported on Ketjen Black (KB), which exhibited excellent performance under a wide pH range (Figure 1g).^[^ ^79^ ^]^ Li et al. prepared multifunctional Co‐IrRu alloy electrocatalysts. Co doping modifies the electronic states of Ir and Ru surfaces, reduced the absorption energy of reaction intermediates, lowered the energy barrier for OER, and changed the *d*‐band center, thereby enhanced the catalytic activity for OER. The OER activity of Co‐IrRu/C‐2 was 248 mV at 10 mA cm^−2^.^[^ ^80^ ^]^ Jiang et al. simultaneously reduced the similarly electronegative Ru^3+^ and Ir^3+^ to form atomically homogeneous Ru_0.5_Ir_0.5_ alloy. The uniform atomic distribution in the alloy enabled Ru atoms to acquire electrons from adjacent Ir atoms, thereby exhibited excellent adsorption ability for intermediates produced during water splitting. The values of Ru_0.5_Ir_0.5_ were 160 mV at 10 mA cm^−2^ in acidic conditions.^[^ ^81^ ^]^

Furthermore, high entropy alloys (HEAs) can modulate the binding energy of reactants in a continuous form with different elemental ratios, providing long‐term stability and excellent catalytic properties.^[^ ^82^ ^]^ Kwon et al. designed a ZnNiCoIrMn HEA, Mn doped into HEA adjusted the electronic structure of Ir sites, the *d*‐band center shifted downward, weakened the adsorption energy with the reaction intermediates, the overpotential of the OER at 10 mA cm^−2^ of ZnNiCoIrMn was 237 mV with a low Ir content (Figure 1h–m).^[^ ^83^ ^]^ Maulana et al. synthesized IrFeCoNiCu‐HEA nanoparticles on carbon paper. The overpotential measured at 10 mA cm^−2^ was 302 mV, which improved the stability of the 12 h compared to the monometallic Ir counterpart without significant phase separation or elemental segregation.^[^ ^84^ ^]^

### 3.3 Metal Oxides

Most commercial acidic OER electrocatalysts at present are still based on Ir‐based oxides, and Ru‐based and other acidic OER electrocatalysts are still in the laboratory stage, a long way from commercialization. Jin et al. prepared chromium‐iridium oxide (Cr_x_Ir_1‐x_O_2_) catalyst. The Cr‐rich catalyst Cr_x_Ir_1‐x_O_2_ showed sustained performance without degradation throughout the long‐term chronoamperometric testing at 10 mA cm^−2^.^[^ ^85^ ^]^ Lu et al. prepared a Co‐doped RuO_2_ nanoparticles anchored on oxidized carbon nanotubes catalyst. Co‐RuO_2_/OCNT exhibited only 260 mV at 10 mA cm^−2^.^[^ ^86^ ^]^ Yan et al. prepared an ultra‐thin nitrogen‐doped carbon nanotube catalyst decorated with RuO_2_ nanoparticles rich in oxygen vacancies. NC@Vo·‐RuO_2_/CNTs‐350 exhibited 170 mV at 10 mA cm^−2^ and excellent stability, making it an effective acid‐stable OER electrocatalyst.^[^ ^87^ ^]^ In addition, perovskite‐like electrocatalysts with fixed components and structures such as single perovskite (ABO_3_), double perovskite (A_2_B_2_O_6_), pyrochlore‐type oxides (A_2_B_2_O_7_), and transition metal oxides in Ruddlesden‐Popper structures is promising acidic OER electrocatalysts due to their low cost, stable structure and adjustable elements.^[^ ^88^ ^]^ You et al. utilized CeO_2_ quantum dots to modify SrIrO_3_ nanosheets. The CeO_2_@SrIrO_3_ heterostructure exhibited excellent OER performance in acidic media, providing a low overpotential of 238 mV at 10 mA cm^−2^ and a durable lifetime of 50 h.^[^ ^89^ ^]^ Liu et al. synthesized a quadruple perovskite‐like oxides. Notably, CaCu_3_Ru_4_O_12_ exhibited the highest activity and stability.^[^ ^90^ ^]^ The OER stability of ABO_3_ materials is insufficient, and pyrochlore oxide Y_2_Ru_2_O_7‐δ_ holds promise as an OER catalyst in acid media. Y_1.75_Co_0.25_Ru_2_O_7‐δ_ catalyst exhibited 275 mV at 10 mA cm^−2^ under acidic condition and only experienced a 3.6% decrease after 2000 cycles (Figure 1n).^[^ ^91^ ^]^

## 4 Optimization of Performance

A significant effort has been made in the design and synthesis of acid OER electrocatalysts to achieve both high activity and stability, while reducing the catalyst loading to lower costs. Various strategies have been explored, such as monodisperse dispersion, component doping, alloying, and structural engineering, to selectively tune the oxidation state, electronic structure, and interaction forces of active sites for effective catalyst screening. These efforts have aimed at the goal of developing excellent electrocatalysts with reduced cost while maintaining high activity and stability.

### 4.1 Improvement of Intrinsic Activity

To enhance the intrinsic activity of electrocatalysts, researchers have adjusted the electronic structure, lattice structure, and complex structures of electrocatalysts by means of alloying, constructing core‐shell structures, and doping with heteroatoms, etc., thereby realizing the improvement of the intrinsic activity of electrocatalysts

#### 4.1.1 Regulation of Electronic Structure

**Figure 2:**
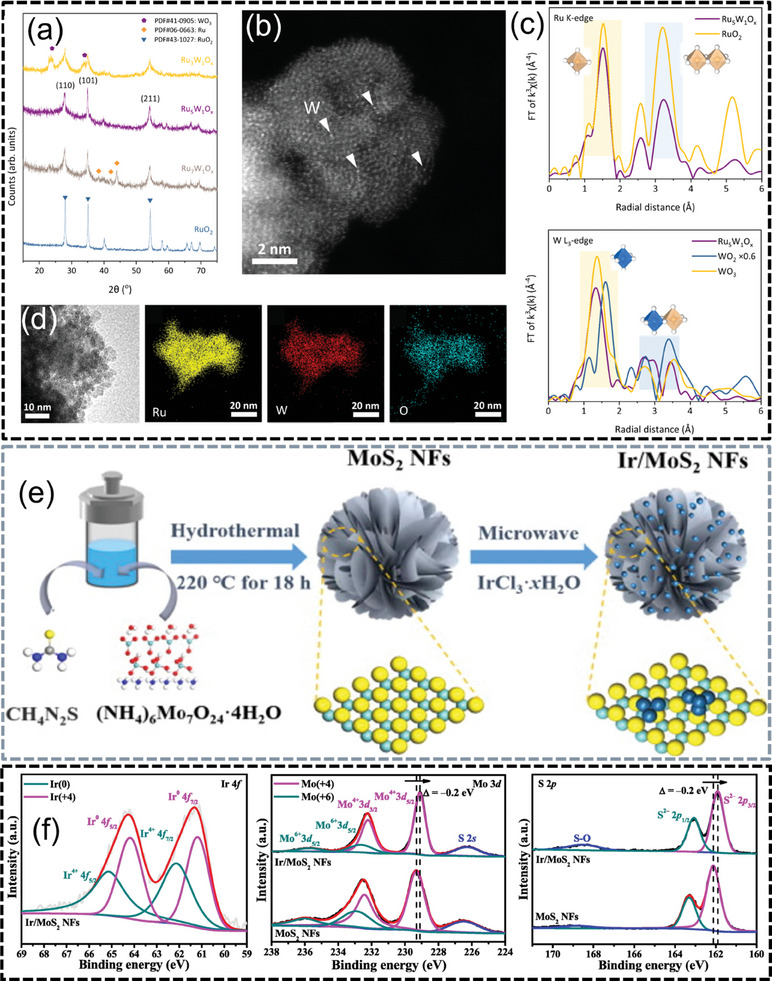
a) The XRD patterns of different electrocatalysts, b) The atomic resolution HAADF‐STEM of Ru_5_W_1_O_x_, c) The *k* ^3^‐weighted Fourier‐transformed extended X‐ray absorption fine structure (FT‐EXAFS) spectra of Ru K‐edge and W L^3^‐edge, d) The HR‐TEM and EDX element mapping of as‐prepared Ru_5_W_1_O_x_. Reproduced with permission.^[^ ^94^ ^]^ Copyright 2022, Springer Nature. e) Schematic illustration of the synthesis of MoS_2_ nanoflowers and Ir/MoS_2_ nanoflowers, f) X‐Ray photoelectron spectroscopy (XPS) spectra of MoS_2_ nanoflowers and Ir/MoS_2_ nanoflowers. Reproduced with permission.^[^ ^95^ ^]^ Copyright 2023, Elsevier.

First‐row 3*d* transition metal oxides and their derivatives, which possess the advantages of high abundance and low cost, have received extensive attention as alternative OER candidates. 4*d*/5*d* noble metal elements have a larger range of electron wave function space, resulting in various electronic structures through the interaction of 3*d* and 4*d*/5*d* orbitals, which are beneficial for improving OER activity (**Figure** **2**).^[^ ^72^, ^92^, ^93^ ^]^

By comparing RuO_2_ nanoparticles doped with Co, Fe, Ni, Mn, and V, as well as their OER activity and stability with that of Ru‐M alloy nanoparticles, it was found that RuO_2_ and doped RuO_2_ nanoparticles exhibited higher activity and stability compared to their respective Ru and Ru‐M alloy counterparts.^[^ ^96^, ^97^ ^]^ Al doping modulated the valence electron structure of the Ru─O hybridized orbitals, thereby modulating the adsorption energy of the oxygen intermediates and thus promoting the formation of O─O bonds. For example, Al‐doped RuIrO_x_ required an overpotential of 178 mV at 10 mA cm^−2^ and maintained high activity for more than 300 h at 100 mA cm^−2^.^[^ ^98^ ^]^ Similarly, by doping Ti atoms onto the surface of IrO_x_/Ir substrates, Ti provided electrons to weaken Ir‐ O interactions through AEM, generating abundant Ir‐O‐Ti groups, which contributed to the excitation of the Ir site and enhanced the OER activity, but the restricted O─O bond formation in the LOM and the expansion of the stabilizing region of Ir species contributed to the enhancement of stability, Ti‐IrO_x_/Ir had an overpotential of 254 mV at 10 mA cm^−2^ and a small Tafel slope of 48.0 mV dec^−1^, reaching a mass activity of 338 A g_Ir_^−1^ at 350 mV.^[^ ^99^ ^]^ The deprotonation of intermediates could be accelerated by introducing strong Brønsted acid sites (WO_x_). The W‐O‐Ru Brønsted acid sites facilitated proton transfer from oxygen intermediates to adjacent bridging oxygen sites, thereby accelerating the bridging oxygen‐assisted deprotonation step in acidic electrolyte. The binary oxide Ru_5_W_1_O_x_ exhibited 227 mV at 10 mA cm^−2^, the overpotential remained at 235 mV with a slow degradation rate after 550 h of stability testing (Figure 2a–d).^[^ ^94^ ^]^ Wang et al. coupled Ir as an active site to 3D MoS_2_ nanoflowers, where MoS_2_ promoted charge reconfiguration and altered the interfacial structure by modulating the electronic structure to allow a more suitable adsorption capacity for H* and HOO*, which required an overpotential of 270 mV to reach 10 mA cm^−2^ (Figure 2e,f).^[^ ^95^ ^]^

#### 4.1.2 Alteration of Lattice Structure

**Figure 3:**
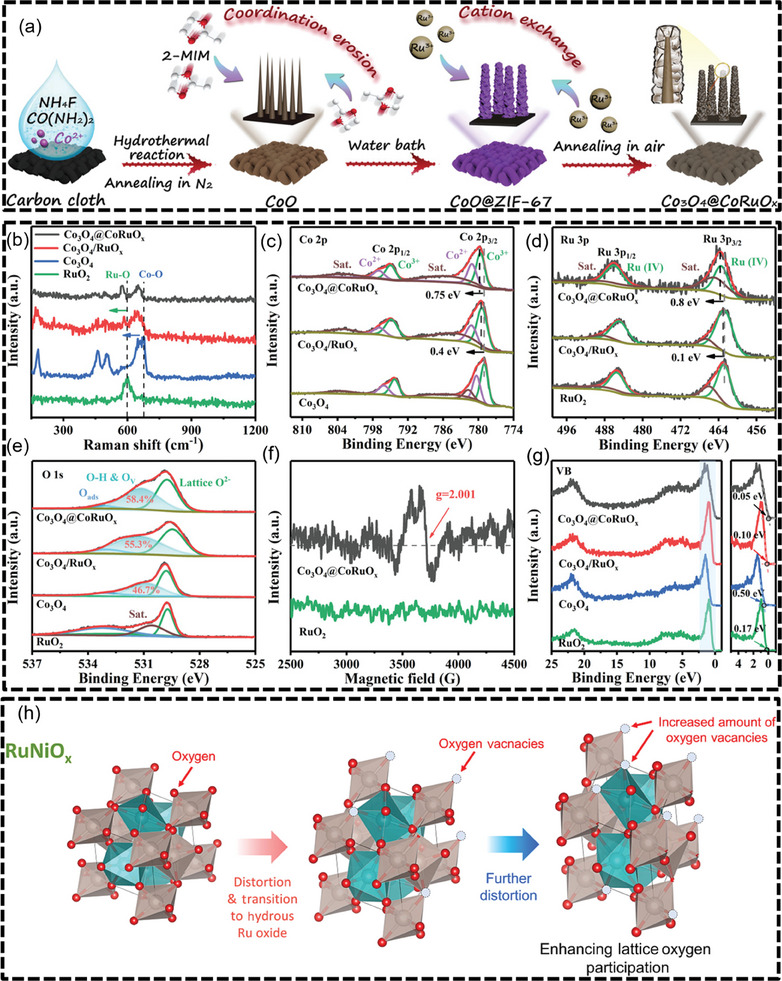
a) Schematic illustration of the synthetic procedure for the core‐shell Co_3_O_4_@CoRuO_x_, b) Raman spectra, c–e) The high‐resolution Co 2p, Ru 3p, and O 1s XPS spectra, f) Electron paramagnetic resonance (EPR) spectra, g) Valence band XPS spectra of Co_3_O_4_@CoRuO_x_ Co_3_O_4_/RuO_x_, Co_3_O_4_, and commercial RuO_2_. Reproduced with permission.^[^ ^100^ ^]^ Copyright 2022, John Wiley and Sons. h) Schematic illustration of the role of Ni introduced in Ru oxide. The structures of the RuNiOx electrocatalysts. Reproduced with permission.^[^ ^49^ ^]^ Copyright 2022, Elsevier.

The OER activity is significantly dependent on lattice properties, and changes in lattice structure can result in alterations in surface electronic structure, OER activation energy, and atomic distances (**Figure** **3**).

Incorporation of transition metals into the Ru oxide lattice can modulate the chemical environment and electronic structure of the Ru sites, thus improving the activity and durability of OER. Gong et al. constructed a cobalt‐doped RuO_x_ framework on Co_3_O_4_ nanocones (Co_3_O_4_@CoRuO_x_). The Co‐doped RuO_x_ lattice introduces oxygen vacancies and lattice shrinkage. The redistribution of the electronic configuration and optimized *d*‐band center equilibrium of the RuO_x_ improves the adsorption energy of the oxygen intermediates, thus lowering the thermodynamic barriers that determine the rate steps. Furthermore, due to the downward shift of the *p*‐band of the lattice oxygen, the peroxide formation and solvation of the Ru species are suppressed, resulting in excellent OER activity and stability of Co_3_O_4_@CoRuO_x_ in acidic electrolytes (Figure 3a–g).^[^ ^100^ ^]^ Ko et al. enhanced the OER catalytic activity by introducing Ni into Ru oxides. The nanostructured RuNiO_x_ electrode exhibited superior OER performance, which achieved an overpotential of 210 mV at 10 mA cm^−2^ and remained stable for 100 h. The introduction of Ni distorts the structure of Ru‐oxide and increases the number of oxygen vacancies at the working potential (Figure 3h).^[^ ^49^ ^]^ Shi et al. directly grew porous RuCuO_x_ nanosheets on carbon paper. Cu doping causes significant distortion of the lattice and alters the electronic characteristics of the Ru sites, thus reducing the adsorption energy of the intermediates and enhancing electrochemical stability and achieving efficient OER activity.^[^ ^101^ ^]^ Huang et al. developed CdRu_2_IrOx nano‐framework with distorted structure, the applied potential led to the distortion of Ru─O, Ir─O, and Ru─M (M═Ru, Ir) bonds in the structure and the deformation of the octahedral structure, which lowered the energy barrier of the rate‐limiting step in the OER process. The CdRu_2_IrO_x_ exhibited an ultra‐low overpotential and an ultra‐long stability.^[^ ^102^ ^]^ Park et al. prepared a manganese oxide nanocatalyst with crystal distortion due to Ir substitution. The partial substitution of tetrahedral Mn(II) in spinel by Ir break the space group symmetry and induced structural distortion and charge distribution changes, which were favorable for oxygen formation and effective charge accumulation, thus controlling the redox properties of Mn and improving the electrocatalytic activity of OER. The overpotential of (Ir_0.04_Mn_0.96_)O_1.45_ at 10 mA cm^−2^ was reduced by 190 mV, and the mass activity at 400 mV overpotential was 2043 A g_Ir_^−1^, which confirmed the long‐term electrochemical stability at 100 mA cm^−2^.^[^ ^103^ ^]^

Similarly, Incorporating Ru or Ir into the lattice of transition metal oxides modulates the chemical environment of Ru or Ir and their adjacent lattice oxygen. Liu et al. used Ir doped ruthenates pyrochlore, where the elongated Ru─O and Ir─O bonds keep Ru and Ir in a low oxidation state compared to RuO_2_/IrO_2_, favoring the formation of the decisive OOH* intermediates. The Y_2_Ru_1.2_Ir_0.8_O_7_ showed a low OER overpotential, low Tafel slope, and high durability. The lattice oxygen provided electrons and charges to stabilize the Ru or Ir, thereby enhancing OER activity and durability.^[^ ^104^ ^]^ For instance, incorporating Ir single‐atom into the lattice structure of transition metal oxides, adjusting the chemical environment of [IrO_6_] octahedra to achieve a balanced distribution of intermediate adsorption energy barriers and more stable low‐valence Ir, which facilitates charge transfer and enhances OER performance. The obtained Ir_0.08_Co_2.92_O_4_ NWs with low Ir content exhibited excellent acidic OER performance with an overpotential of only 189.5 mV and excellent durability for over 100 h at 10 mA cm^−2^.^[^ ^73^ ^]^

#### 4.1.3 Configuration of Complex Structure

**Figure 4:**
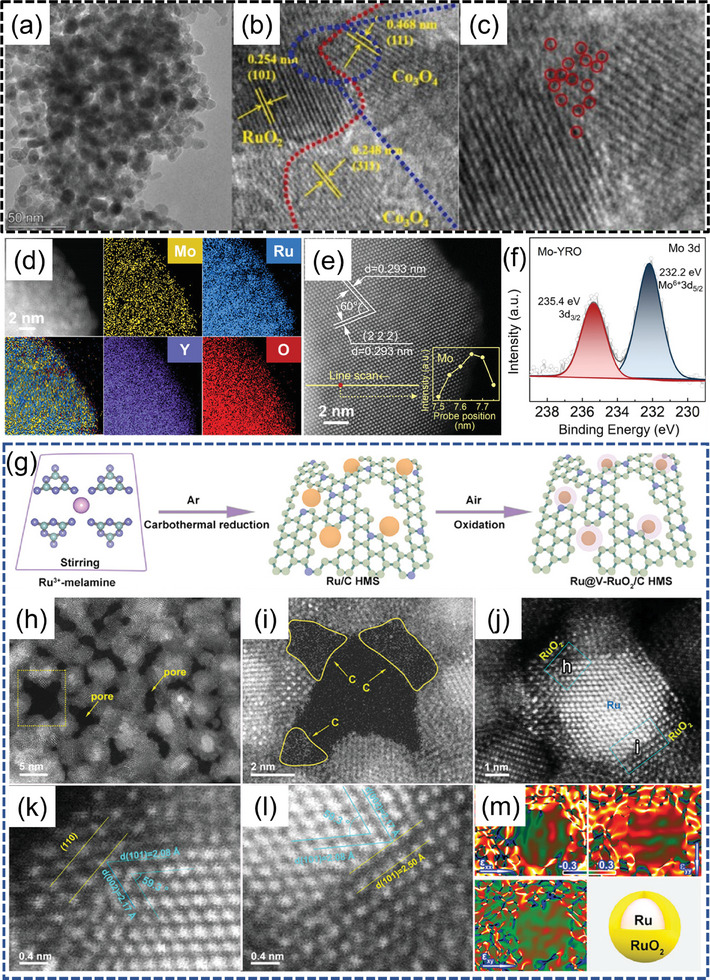
a) HRTEM of RuO_2_@Co_3_O_4_ heterojunction, Magnified TEM of b) RuO_2_@Co_3_O_4_ hetero‐interface, c) Interfacial oxygen vacancies. Reproduced with permission.^[^ ^106^ ^]^ Copyright 2023, Elsevier. d) STEM‐EDS elemental, e) HAADF‐STEM, f) High‐resolution Mo 3d XPS spectrum of Mo‐YRO. Reproduced with permission.^[^ ^108^ ^]^ Copyright 2023, John Wiley and Sons. g) Schematic illustration of the formation for Ru@V‐RuO_2_/C HMS, h–l) aberration‐corrected HAADF‐STEM, m) the corresponding strain maps along *ε_xx_*, *ε_yy_*, *ε_xy_* processed by the GPA algorithm, as well as the core‐shell model. Reproduced with permission.^[^ ^110^ ^]^ Copyright 2023, John Wiley and Sons.

Well‐defined interface structures can modulate the electronic structure of electrocatalysts, accelerate electron transfer, significantly promote the adsorption of necessary reaction intermediates and enhance electrocatalytic activity.^[^ ^41^, ^105^ ^]^ Spontaneous electron transfer occurs at the heterointerfaces, and the resulting charge redistribution benefits the electron transfer reactions at the electrode surface. The interaction between high electrical conductivity of crystal phases and abundant unsaturated coordination sites in amorphous phases is crucial for constructing heterogeneous interface structures (**Figure** **4**). Jiang et al. prepared a RuO_2_@Co_3_O_4_ electrocatalyst with tuneable heterogeneous interfaces and enhanced vacancy effects. Different ratios of Ru and Co could adjust the number of heterogeneous interfaces and the concentration of interface oxygen vacancies between RuO_2_ and Co_3_O_4_. The heterointerface with strong electron coupling between Co_3_O_4_ and RuO_2_ accelerated kinetic reactions and improved the stability of catalyst. The oxygen vacancies directly participated in the dissociation of H_2_O and regulated the position of the *d*‐band center and the adsorption strength of critical intermediates, thereby enhancing the catalyst's activity (Figure 4a–c).^[^ ^106^ ^]^ Liu et al. introduced Co into the non‐crystalline/crystalline ZnRuO_x_ nanocage to regulate the electronic environment of Ru, optimize the adsorption energy of intermediate species, and expose more active sites. Through the synergistic coupling effect between the non‐crystalline/crystalline hetero‐phase structure, the addition of Co prevented the overoxidation of Ru, achieving a leap from single‐function to multi‐function electrocatalysts.^[^ ^107^ ^]^ Liu et al. implanted MoO_x_ moieties onto nanostructured Y_2_Ru_2_O_7‐δ_ to obtain Mo‐YRO catalyst. Unlike intramolecular charge transfer regulating the electronic structure, the heterostructure Mo‐O‐Ru interface facilitated efficient intermolecular electron transfer from [RuO_6_] to MoO_x_, eliminating the bandgap by inducing Ru 4d delocalization and band alignment rearrangement. The aberration of [RuO_6_] is mitigated by the shortening of the Ru─O bond length and the increase of the Ru─O─Ru bond angle in the presence of MoO_x_. This tailored electronic and geometric structure boosted OER performance, resulting in a small overpotential of 240 mV at 10 mA cm^−2^. Moreover, the electron‐accepting MoO_x_ moieties provided a more electronegative surface, suppressing the dissolution of Ru ions and stabilizing the electrochemical activity (Figure 4d–f).^[^ ^108^ ^]^ Zheng et al. grafted Ir nanoparticles onto antimony tin oxide nanocrystalline to construct Ir‐Sn pair‐site electrocatalyst (Ir‐Sn PSC) with heterogeneous structures. The formation of Ir‐Sn duplexes as well as the resulting strong electronic interactions greatly reduced the *d‐*band holes of Ir during the OER process, inhibited their overoxidation, and led to a much higher corrosion resistance of the electrocatalysts. As a result, the optimized Ir‐Sn PSC has a high mass activity of 4.4 A mg_Ir_^−1^ at an overpotential of 320 mV and good long‐term stability. The PEMWE had a current density of 2 A cm^−2^ at 1.711 V and a low degradation rate in accelerated aging tests.^[^ ^109^ ^]^

Core/shell configurations can synergistically control the adsorption/desorption kinetics of intermediates by strain and ligand effects to optimize catalyst activity. The strain effect achieved by core‐shell structures is short‐range and requires the cooperative interaction of multiple atomic layers, thus requiring strict control over the structure. Li et al. armored sub‐nanometer RuO_2_ as a shell onto the interconnected Ru clusters/carbon hybrid micro‐sheet (C HMS), the armored RuO_2_ maintained better conductivity due to the presence of Ru cores and facilitated electron transfer. The Ru@V‐RuO_2_/C HMS catalyst showed significantly low overpotential of 176 mV at 10 mA cm^−2^ under acidic conditions (Figure 4g–m).^[^ ^110^ ^]^

### 4.2 Increasing the Number of Active Sites

The catalytic processes generally occur on the surface of electrocatalysts. In addition to enhancing intrinsic activity, the abundance of active sites can be increased through rational structural design, such as improving carrier morphology, coordination environment, dispersion density and more favorable shaping strategies, etc.^[^ ^59^ ^]^

#### 4.2.1 Regulation of Morphology and Dispersion State

Surface morphology and dispersion state plays a critical role in activity and durability. Electrocatalysts with strong metal bonds and high surface energy are difficult to be uniformly distributed and density controlled at high temperatures.^[^ ^111^, ^112^ ^]^ Reducing particle size and increasing dispersion can increase the electrochemical active surface area, improve the specific activity of the catalyst, and enhance the utilization efficiency of precious metals.^[^ ^113^ ^]^ To reduce catalyst particle size and avoid aggregation, surface morphology can be controlled to prepare nano‐sheets, nanoparticles, clusters, hollow nanomaterials, etc., which enhance OER activity.^[^ ^114^ ^]^ Furthermore, appropriate pore size not only facilitates mass transfer but also minimizes bubble growth, accelerates separation, and prevents active site clogging.^[^ ^115^ ^]^

Metal‐organic frameworks (MOFs) possess the highly porous structure and the existence of densely and uniformly dispersed catalytic active sites.^[^ ^116^, ^117^, ^118^ ^]^ Covalent Organic Polymers (COPs) are suitable catalyst support materials with adjustable pore size, controllable components, and excellent thermal stability. Shao et al. prepared surface density‐controllable nano‐clusters (NCs) with the help of COP. COP provided continuous anchoring sites, hindering atom migration, while the thermally stable porous structure provided a size‐restricted skeleton, limiting the particle size of Ir NCs and improving surface atomic utilization. The synthesized Ir‐COP exhibited an overpotential of 242 mV at 10 mA cm^−2^ in acidic conditions for OER. Additionally, the strong interaction between Ir NCs and nitrogen‐doped carbon layers in Ir‐COP resulted in outstanding stability and significant overall water‐splitting performance in both acidic and alkaline media.^[^ ^119^ ^]^ Co_3_O_4_ has a highly porous network, doped with Ru, the deformation induced by the modulation of the electronic structure provided more active sites and facilitated the penetration of the electrolyte, which enhanced the electron transport on the exposed active metal sites. Ru‐Co_3_O_4_ exhibited a high durability of 16.5 h at a lower overpotential of 365 mV driving a current density of 10 mA cm^−2^ in an acidic medium. Moreover, Hollow carbon nanocages with mesopores, macropores, and micropores are highly porous with high specific surface area, easy doping and modulation, which can greatly promote the exchange and transfer of substances in liquid‐solid and gas‐solid electrocatalytic reactions and effectively accommodate the strain relaxation during energy storage processes.^[^ ^120^ ^]^ Mesoporous silica templates such as SBA‐15, KIT‐6, and hexagonal copper sulfide nanosheets were used to synthesize efficient OER electrocatalysts with ordered mesoporous nanostructures.^[^ ^121^, ^122^ ^]^

#### 4.2.2 Improvement of the Carrier Environment

The catalyst is strongly influenced by the carrier, and the strong interaction between the metal sites and the carrier helps to enhance the dispersion of the active sites, prevents the aggregation of the metal sites on the carrier, safeguards the exposure of the metal sites, and facilitates the transfer of electrons between the carrier and the metal sites, resulting in improved electrochemical activity and stability.^[^ ^123^, ^124^, ^125^, ^126^ ^]^

To enhance the OER activity while reducing the loading of precious metals, the electronic structure of electrocatalysts is usually improved by introducing second sites (e.g., doped heteroatoms) on the carriers, and these catalytic materials exhibit rich and tuneable electronic structure characteristics, which presents a promising prospect in the field of OER.^[^ ^127^ ^]^ By introducing heteroatoms such as nitrogen, boron, phosphorus, and sulfur, the surface properties of materials can be optimized.^[^ ^128^, ^129^, ^130^, ^131^, ^132^, ^133^, ^134^ ^]^ Joshi et al. explored the influence of flake size of nitrogen‐doped reduced graphene oxide (N‐rGO) on the deposited IrO_2_ for OER electrocatalytic activity.^[^ ^135^ ^]^ Yao et al. anchored Ir nanoparticles on N‐rGO using a one‐pot hydrothermal method, preparing electrocatalysts (Ir/N‐rGO) with a size of about 2 nm. Compared with Ir/rGO without N doping, Ir/N‐rGO exhibited higher current density and lower onset oxidation potential, with an overpotential of only 260 mV and a Tafel slope of 60.4 mV dec^−1^. The ultra‐small Ir nanoparticles and N doping on the graphene surface generated a large number of defects, exposing more electrocatalytic active sites of the catalyst, making it suitable as a bifunctional electrode for overall water splitting.^[^ ^136^ ^]^ Ding et al. immobilized Co clusters containing single Ir atoms on an N‐C support to prepare a multiphase single‐atom cluster catalyst Co_n_Ir_1_/N‐C. The distance between the Ir single atoms and Co cluster was optimized to be ≈8 Å, which improved the configuration of critical intermediates. The results showed that the OER activity of ConIr_1_/N‐C was significantly higher than that of Co cluster electrocatalysts (Co_n_/N‐C), with an overpotential 107 mV lower than that of Co_n_/N‐C at 10 mA cm^−2^.^[^ ^137^ ^]^ Zhao et al. anchored IrO_2_ NPs on exfoliated h‐BN nanosheets, and the epitaxial interface between IrO_2_ and BN scaffold further stabilized the high‐valence active species, with an overpotential of 390 mV at 100 mA cm^−2^. Compared with bare IrO_2_ NPs or IrO_2_/C, IrO_2_/BN showed significantly improved OER stability in acidic media.^[^ ^138^ ^]^

### 4.3 Enhancement of Stability

Apart from increasing catalytic activity and reducing catalyst cost, the long‐term stability of OER electrocatalysts is of paramount importance. More and more efforts have been devoted to the development of strategies to improve the catalytic stability of OER electrocatalysts, especially under acidic conditions, where the mechanisms leading to catalyst deactivation are complex and numerous, such as dissolution or peroxidation of the active sites, changes in the crystal structure, reconstruction of the surface structure, clogging of bubbles on the surface of the electrodes, and detachment of the catalyst from the electrodes. Thus, factors such as the oxidation state of the active metal, crystal structure, oxygen vacancies, dopants, characteristics of the substrate electrode, and electrolyte conditions can affect the stability of OER electrocatalysts.^[^ ^139^, ^140^ ^]^ Later we will highlight the main OER catalyst stability enhancement approaches available.

#### 4.3.1 Prevention of Active Site Dissolution/Overoxidation

**Figure 5:**
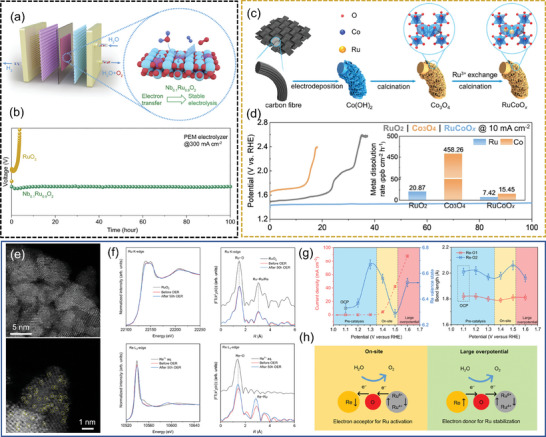
a) Nb_x_Ru_1−x_O_2_ in a three‐electrode system and PEM electrolyzer, b) The corresponding stability test of the PEM electrolyzer at 300 mA cm^−2^. Reproduced with permission.^[^ ^144^ ^]^ Copyright 2023, Elsevier. c) Synthesis of the RuCoO*_x_*, d) Acidic OER performance of chronopotentiometry curves of the RuO_2_, Co_3_O_4_, and RuCoO*_x_* at 10 mA cm^−2^. Reproduced with permission.^[^ ^65^ ^]^ Copyright 2023, American Chemical Society. e) Aberration‐corrected HAADF‐STEM image of Re_0.06_Ru_0.94_O_2_ after 50 h OER, f) Ru K‐edge and Re L_3_‐edge XANES spectra and FT *k* ^2^‐weighted EXAFS signals for RuO_2_, Re_0.06_Ru_0.94_O_2_ before and after 50 h OER, g) Change in Re valence state and OER current as a function of applied potential and change in bond length for Re‐O1 and Re‐O2 coordination shells, h) Schematic for dynamic electron transfer in Re_0.06_Ru_0.94_O_2_. Reproduced with permission.^[^ ^147^ ^]^ Copyright 2023, Springer Nature.

Ir can be excessively oxidized to soluble IrO_4_2^−^, Ru is susceptible to over‐oxidation to volatile RuO_4_ or soluble RuO_5_^2−^ at high potentials, leading to a decrease in its stability.^[^ ^141^, ^142^ ^]^ The introduction of metallic/non‐metallic heteroatoms into the electrocatalysts by chemical doping strategies modulates their lattice structures, electronic structures, and coordination environments to inhibit the over‐oxidation and dissolution of the noble metal active components at high potentials and current densities, as well as to improve the adsorption of reactive oxygen intermediates to the active sites (**Figure** **5**).^[^ ^143^ ^]^ Liu et al. introduced a high‐valence transition metal Nb into RuO_2_, and Nb‐RuO_2_ transferred electrons from Nb to Ru through bridging oxygen, increasing the electron density. The formation of Nb^5+^ in RuO_2_ inhibited the dissolution of Ru, enhanced the Ru─O bonds in RuO_2_, prevented the formation of soluble high‐valence Ru, and Nb_0.1_Ru_0.9_O_2_ remained stable for 360 h at a current density of 200 mA cm^−2^ (Figure 5a,b).^[^ ^144^ ^]^ Li et al. moderately lowered the *d*‐band center energy level of Ru in Nd‐doped RuO_2_, balancing the adsorption and desorption of oxygen intermediates. The prepared Nd_0.1_RuO_x_ with a higher proportion of Ru^4+^ significantly suppressed the dissolution of Ru in acidic electrolyte and exhibited good durability.^[^ ^145^ ^]^ Liang et al. used Ru to selectively replace the Co site on octahedral Co_3_O_4_, and prepared RuCoO_x_ with highly symmetric Ru─O─Co synergistic coordination and strong electronic coupling effect, the enriched electrons around the Co atom can transfer the electrons through the bridging oxygen bond, which can inhibit the over‐oxidation and solvation of the active Ru and Co, and can be stabilized for 100 h at 10 mA cm^−2^ (Figure 5c,d).^[^ ^65^ ^]^ Zhang et al. synthesized orthorhombic (Ru, Mn)_2_O_3_ oxide solid solutions by replacing some of the Mn in Mn_2_O_3_ with Ru. The lower valence state of Ru in (Ru, Mn)_2_O_3_ provides higher solubility resistance, and the atomic ratio of Ru is higher than that of rutile RuO_2_, resulting in better activity, and a good stability of the OER at the same current for at least 40 h in an acidic medium.^[^ ^146^ ^]^

Although the doping of some heteroatoms can achieve an enhancement of the OER stability of the catalyst in acidic media, the enhancement is still insufficient and most of them can only achieve stability at lower overpotential. Once the overpotential exceeds a certain range, over‐oxidative dissolution of Ru occurs as well. The addition of a continuously variable metal such as Re (oxidation state can be from −3 to +7) can alleviate this phenomenon to some extent. By doping Re into RuO_2_, adaptive active sites with dynamic electron transfer can be constructed and the effect of stabilizing both lattice oxygen and low‐valent metal active sites can be achieved. Jin et al. doped Re into RuO_2_ and obtained a Re_0.06_Ru_0.94_O_2_ catalyst. At small overpotentials, Re gained electrons from Ru, and the valence state of Re increased with increasing oxidation potential, activating Ru sites. At large overpotentials, Re feedbacked electrons to prevent excessive oxidation of Ru active sites and the formation of H_2_RuO_5_ species. The Re doping agent acted as a dynamic electron reservoir to modulate the electronic structure of Ru. Re_0.06_Ru_0.94_O_2_ exhibited 190 mV at 10 mA cm^−2^ and a Tafel slope of 45.5 mV dec^−1^. Moreover, Re_0.06_Ru_0.94_O_2_ maintained a stable potential after continuous testing for 200 h at a constant current density of 10 mA cm^−2^ (Figure 5e–h).^[^ ^147^ ^]^

#### 4.3.2 Reduction of the Collapse of the Crystal Structure

**Figure 6:**
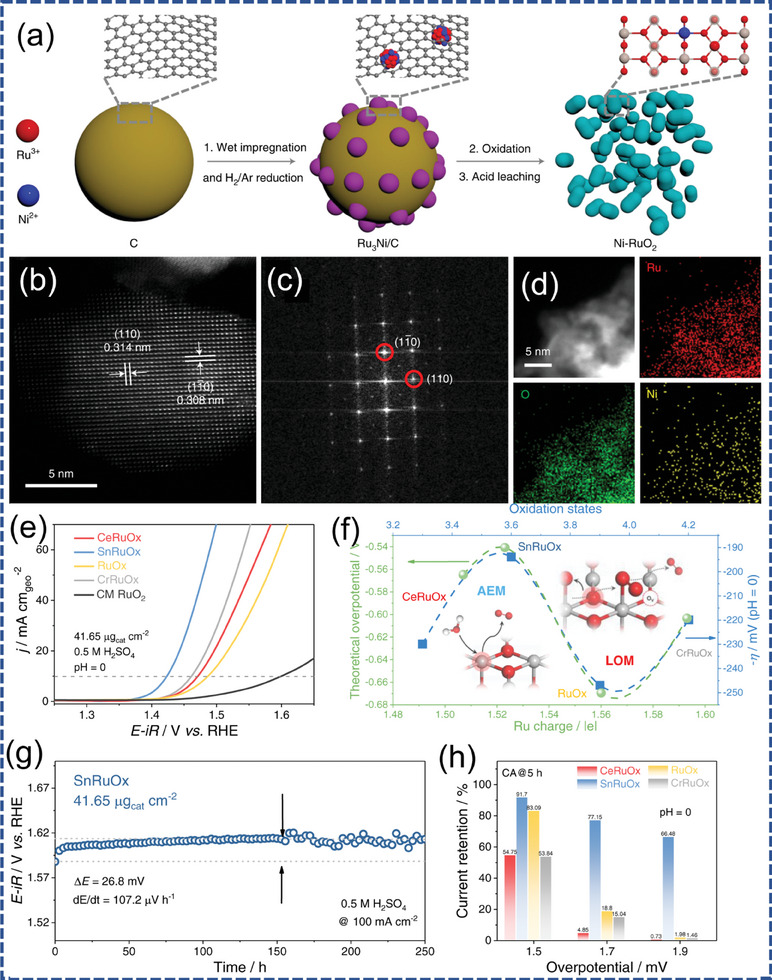
a) Schematic illustrating the synthesis of Ni‐RuO_2_, b) HADDF‐STEM, c) Corresponding fast Fourier transform pattern, and d) EDS mapping of the Ni‐RuO_2_ catalyst. Reproduced with permission.^[^ ^149^ ^]^ Copyright 2022, Springer Nature. e) Normalized LSV curves of MRuO_x_, f) The variation of apparent overpotential at 10 mA cm^−2^ with Ru oxidation states, g) Chronopotentiometry curve of SnRuO_x_ nanocatalyst operated at 100 mA cm^−2^ during the 250 h test, h) Current retention of MRuO_x_ after chronoamperometry test at various potentials for 5 h. Reproduced with permission.^[^ ^152^ ^]^ Copyright 2023, Springer Nature.

To reduce crystal structure collapse, the doping of additives into the IrO_2_/RuO_2_ lattice results in stronger hybridization between the 5*d* and O 2*p* orbitals, thereby minimizing the dissolution of noble metals. However, excessive hybridization between the transition metal d and O 2*p* orbitals may lead to surface amorphization or even crystal structure collapse during the OER process. Therefore, it is necessary to search for dopants with high electrochemical stability, multiple valence states, and adjustable oxygen capacity (**Figure** **6**). The ion size of Re is like that of Ir, allowing for a larger spontaneous substitution energy. By incorporating Re with low crystallinity into the IrO_2_ crystal, the multiple valence states, and adjustable oxygen content of Re not only inhibit the dissolution of Ir but also maintain the original crystal structure of IrO_2_. The optimized catalyst Re_0.1_‐IrO_2_ exhibited a low overpotential of 255 mV at 10 mA cm^−2^ and high stability for OER under acidic conditions for 170 h.^[^ ^148^ ^]^ The electrocatalysts directly demetallize under acidic conditions, leading to the collapse of the crystal structure and decreased OER stability. The original RuO_2_ exhibited poor acidic OER stability and degraded upon continuous operation in a short period of time. However, the addition of Ni not only stabilized the surface Ru but also significantly stabilized the RuO_2_ lattice, resulting in a remarkable increase in the density of active sites. The Ni‐stabilized RuO_2_ (Ni‐RuO_2_) had a 214 mV at 10 mA cm^−2^, with a Tafel slope of 42.6 mV dec^−1^. After continuous operation for 200 h, the potential of Ni‐RuO_2_ increased negligibly. When applied as an anode in a PEMWE cell under a water decomposition current of 200 mA cm^−2^, it exhibited stability exceeding 1000 h, demonstrating its potential for practical applications (Figure 6a–d).^[^ ^149^ ^]^ Transition metal electrocatalysts can induce the rupture of relatively weak Si─C bonds in COP1‐T, resulting in the formation of Ru─Si bonds in Si‐RuO_x_@C, with Si incorporated into the RuO_2_ lattice. Si‐RuO_x_@C maintained constant OER for 100 h in acidic electrolyte, showing stable CV energy even after 27 000 cycles.^[^ ^150^ ^]^ Song et al. encapsulated a small amount of ruthenium nanoclusters into nitrogen‐vacancy‐rich g‐C_3_N_4_, and the introduction of many nitrogen‐containing vacancies effectively modulated the *d‐*band center, the intermediate binding energies, and the structural stability. The optimized Ru NCs/VN‐C_3_N_4_ has a unique porous structure and rich defects, which greatly exposes the active sites, with ultra‐high mass activity and better total hydrolysis performance.^[^ ^151^ ^]^ The instability of the catalyst is closely related to the difficulty of Ru─O bond breakage, which depends on the formation energy between oxygen vacancies and Ru vacancies. Shi et al. controlled the M‐O‐Ru structure by changing the chelating elements around the RuO_6_ subunit to successfully regulate the reaction mechanism, namely, whether lattice oxygen participates in the reaction, and successfully regulated the activity and stability of the adsorbate evolution reactions. SnRuO_x_ solid solution with a local Sn‐O‐Ru structure followed AEM and had an appropriate Ru─O bond strength. The SnRuO_x_ catalyst exhibited high intrinsic activity, with an increase of only 26.8 mV in overpotential after testing for 250 h at 100 mA cm^−2^. In a PEM electrolyzer, during a 1300 h durability test at 1 A cm^−2^ (Figure 6e–h).^[^ ^152^ ^]^

#### 4.3.3 Rational Utilization of Surface Reconstruction

At the initial stage of OER, due to the high oxygen evolution potential, surface reconstruction occurred on the OER catalyst, resulting in the formation of transition metal‐based (oxy)hydroxides or bulk materials. These formed (oxy)hydroxides on the surface became the actual catalytically active species, leading to a change in the active sites of OER and a decrease in stability.^[^ ^153^ ^]^ The rutile structure possesses advantages in terms of dense stacking, shared edges, and tuneable electronic, which help to maintain the coordination structure and resist the dissolution of active sites in strong acidic environments.^[^ ^154^ ^]^ Therefore, by utilizing the shared edges of metal‐oxygen octahedra, an active structure of rutile‐like amorphous metal oxides can be formed on the catalyst surface, achieving low‐loading, long‐lasting acid OER electrocatalysts. This is currently the main research direction for reducing the influence of surface reconstruction.

**Figure 7:**
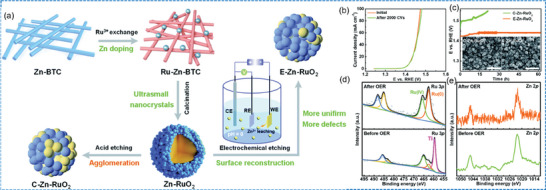
a) Illustration of the synthetic route for the E‐Zn‐RuO_2_ and C‐Zn‐RuO_2_, b) LSV of E‐Zn‐RuO_2_ before and after 2000 CV cycles, c) Stability of C‐Zn‐RuO_2_ and E‐Zn‐RuO_2_ at 10 mA cm^−2^, d) Ru 3p and e) Zn 2p of E‐Zn‐RuO_2_ before and after acidic OER. Reproduced with permission.^[^ ^157^ ^]^ Copyright 2023, Springer Nature.

Ir‐based perovskite (ABO_3_) has multifunctional coordination sites and adjustable oxidation states. However, under strong oxidative electrocatalytic conditions, the A‐site cations are prone to leaching from the perovskite lattice into the acidic electrolyte, leading to the destruction of the electrochemically active IrO_6_ octahedral network, generation of oxygen vacancies, and accelerated dissolution of Ir, resulting in poor durability and severe surface reconstruction in acidic media. Ir‐rich double perovskite Sr_2_CaIrO_6_ with Ir atoms in a high oxidation state (Ir^6+^/^5+^), notable reconstruction occurred on the surface of the perovskite due to the dissolution of Ca and Sr cations during the first OER cycle. This dissolution led to the formation of an outer layer rich in Ir, with Ir atoms in oxidation state (Ir^3+^/^4+^), while the original perovskite framework remained intact and the voids were filled with H_3_O^+^ molecules. The reconstructed surface consisted of short‐range ordered regions of iridium oxide octahedra with short edges and shared corners, resulting in Sr_2_CaIrO_6_ exhibiting high OER activity and only observing a 15% decrease in activity after 5000 cycles.^[^ ^155^ ^]^ In addition to, Sr^2+^ exchange with H^+^ to form protonated γ‐SIO (H‐γ‐SIO), which maintained the open framework structure of γ‐SIO but presented an asymmetric structure. During the OER process, the open‐framework structure did not reconstruct into the commonly observed amorphous IrO_x_H_y_ phase in other iridate‐derived electrocatalysts but restructured into ultra‐small, surface hydroxylated (200)‐oriented rutile nanocatalysts. The high activity and high‐density Ir sites, combined with the ultra‐small nanocrystal size, contributed to the excellent OER catalytic activity, providing a low overpotential of only 200 mV at 10 mA cm^−2^ and an ultra‐long lifetime of 1080 h.^[^ ^156^ ^]^ Chai et al. etched Zn‐doped RuO_2_ nanorods (E‐Zn‐RuO_2_), where Zn doping resulted in a change in the electronic structure and a shortening of the Ru─O bond, and the E‐Zn‐RuO_2_ underwent pre‐oxidation and irreversible surface remodelling to form a stable and active surface, which could be operated stably for 60 h at 10 mA cm^−2^ (**Figure** **7**).^[^ ^157^ ^]^ Ru induced the reconfiguration of the Co_2_P surface into Ru‐ RuP_x_‐Co_x_P hollow polyhedral, and the introduction of in situ Ru not only modulated the M─P bonds, but also reconstructed the electronic structure of the surface, increased the density of states at the Fermi energy level, reduced the adsorption energy gap between OH and OOH intermediates, and enhanced the activity of conventional Co_2_P.^[^ ^158^ ^]^ OER electrocatalysts based on the LOM mechanism are less stable in acids compared to AEM due to the oxidation of lattice oxygen causing severe surface reconstruction followed by dissolution in the electrolyte, leading to a loss of catalytic stability. Wang et al. employed GO as a template and used ion exchange adsorption to introduce Rh into the RuO_2_ lattice, resulting in the preparation of Rh‐RuO_2_/G. Stable oxygen vacancies were generated to compensate for the effective negative charge of the doped low‐valence cations. Through the synergistic control strategy of Rh doping and surface oxygen vacancies, the Rh‐RuO_2_/G catalyst exhibited a low overpotential and the generated oxygen vacancies served as active adsorption sites without participating in the generation of O_2_, leading to stable regeneration. The catalyst showed long‐term stability for 700 h at 50 mA cm^−2^ and 500 h at 100 mA cm^−2^.^[^ ^159^ ^]^

#### 4.3.4 Regulation Interference from the External Environment

The bubble escape rate, the binder, and the type of collector all have an effect on the stability of the OER. These effects have been widely noticed in alkaline OER processes and have not received enough attention in acidic OER processes.

The abundant nano‐scale porous structure can accelerate gas release and rapidly recover active sites, improving the generation and detachment of bubbles on the electrode surface and achieving efficient and sustained catalytic reactions of the catalyst.^[^ ^115^ ^]^ Tian et al. prepared a Yolk‐Shell Structured metal alloy oxide ZnCo‐RuO_2_/C with a porous shell structure exposing abundant active sites, which facilitates OER mass transfer with an overpotential of only 180 mV at 10 mA cm^−2^ and a Tafel slope of 63 mV dec^−1^.^[^ ^160^ ^]^ Glassy carbon electrodes (GC) are frequently used electrode platforms in electrocatalytic activity tests due to their advantages such as high temperature resistance, high hardness, and low resistance. However, their drawback is the slow removal of bubbles from the electrode surface, limiting the OER kinetics. By hydrophilizing the working area of the electrode to reduce the size of bubbles, the OER performance can be significantly enhanced. Kirti et al. used a plastic chip electrode (PCE) as the working electrode, which is a dual‐component polymer composed of graphite and polymethyl methacrylate. The two‐phase material with different wettability can inhibit the formation of large‐sized bubbles, helping to reduce electrode passivation.^[^ ^161^ ^]^

Most electrocatalysts exist in powder form and are typically fixed on electrodes and in contact with electrolytes through a binder, such as Nafion, in testing and electrochemical setups. This leads to the loss of electrode conductivity and active sites and is prone to catalyst detachment due to weak cohesive strength. Therefore, it is imperative to enhance the adhesion between the support and the catalyst.^[^ ^162^ ^]^ Carbon cloth (CC), which is inexpensive, highly conductive, and flexible, is commonly used as a support material for various energy storage materials. You et al. achieved in situ growth of Ru nanoparticles on carbon cloth (Ru‐G/CC) by solvent‐thermal method with glycerol assistance. Ru‐H_2_O/CC‐350 exhibited high electrocatalytic OER activity with an overpotential of 270 mV and a Tafel slope of 63 mV dec^−1^, surpassing commercial RuO_2_.^[^ ^163^ ^]^ Zhang et al. obtained a 3D needle‐like Ru‐doped Ni/Co oxide (Ru‐NiO/Co_3_O_4_) array on CC substrate, which exhibited excellent catalytic activity and stability for OER, ORR, and HER under alkaline conditions. The 2% Ru‐NiO/Co_3_O_4_ electrode achieved 100 mA cm^−2^ OER at only 269 mV overpotential.^[^ ^164^ ^]^ Yang et al. achieved in situ growth of ^SA^Ru/NiFe LDH on 3D porous nickel foam substrate with excellent conductivity and large specific surface area. Compared to ^SA^Ru/NiFe LDH (8 h), ^SA^Ru/NiFe LDH@NF exhibited increased stability for 16 h at a current density of 10 mA cm^−2^.^[^ ^165^ ^]^

## 5 Summary and Outlook

Extensive research has been conducted on noble metal‐based acidic OER electrocatalysts, but continuous investigation was still required.

### 5.1 Enhancement of Atomic Utilization

The high cost and limited reserves of noble metal electrocatalysts necessitate minimizing their consumption while maintaining catalytic performance. Significant progress has been made in the development of single‐atom electrocatalysts, heteroatom‐doped electrocatalysts, and alloy electrocatalysts, which have demonstrated improved intrinsic activity and the exposure of more active sites. However, there is still considerable room for improvement in terms of stable and long‐lasting catalyst usage and recovery.

### 5.2 Development of Multifunctional Electrocatalysts

Superior electrocatalysts require high intrinsic activity, abundant active sites, efficient electron, and mass transfer, as well as high chemical and structural stability. Currently, most Ir‐based and Ru‐based electrocatalysts are focused on single or bifunctional catalysis, which is crucial for electrochemical water splitting. However, achieving multifunctionality with a single catalyst remains challenging. Therefore, it is necessary to explore innovative strategies that synergize intrinsic active sites, electron transfer, mass transfer and gas evolution, mechanical and chemical durability, to better meet the requirements of noble metal‐based electrocatalysis in practical water splitting. Additionally, current noble metal‐based electrocatalysts mainly focus on OER performance under acidic conditions, while there is still a need for specific ion exchange membranes with high durability in strong acid or alkali electrolytes, acid/alkali‐stable electrocatalysts, and corrosion‐resistant fuel cells. In comparison, operating devices under neutral or near‐neutral conditions are more environmentally friendly and cost‐effective. Hence, active efforts should be made to develop highly efficient and stable electrocatalytic materials in neutral electrolytes.

### 5.3 Characterization of the Catalytic Mechanism

Advanced characterization techniques are required to elucidate catalytic mechanisms. Despite the excellent OER performance of precious metal‐based nanomaterials, there is still a lack of in‐depth research on catalytic site determination, surface reconstruction, surface evolution, and interactions between different components. Developing advanced in situ techniques such as XPS, Synchrotron X‐ray absorption spectroscopy (XAS), infrared spectroscopy, and Raman spectroscopy is necessary to monitor the chemical composition of electrocatalysts, changes in the oxidation states of metal ions, nanoscale structure/surface dissolution, and real‐time atomic‐scale variations. This would facilitate the observation of catalyst surface evolution, active sites, and adsorption of reaction intermediates, thereby enhancing the understanding of the reaction process. Furthermore, integrating experimental results with DFT calculations would provide a better understanding of the electrochemical process of OER electrocatalysts.

### 5.4 Determination of Catalyst Evaluation Criteria

Both the AEM and LOM can describe the activity trends of electrocatalysts. Currently, the overpotential at 10 mA cm^−2^ (*η_10_*) and Tafel slopes are the most commonly used parameters to evaluate catalyst activity, while parameters such as exchange current density, TOF, and stability are also frequently employed (**Tables** **1** and **2**). However, important parameters are only reported in a few articles, such as electrochemical active surface area (ECSA) and mass activity (MA). Determining ECSA is a key challenge that requires tracking the actual behavior of active sites and clarifying the observed performance. Merely considering MA based on mass factors cannot provide a thorough characterization of electrocatalysts, as it is influenced by changes in specific activity (SA) of active sites or improvements in dispersion of active phases on supports/substrates. Additionally, there is a lack of standardized methods for benchmark testing the performance of OER electrocatalysts. Factors such as catalyst mass loading, pre‐treatment processes, ink preparation, and electrode fabrication methods are not consistent, leading to unfair comparisons of various catalyst performances. Therefore, standardized experimental protocols are crucial for the fair evaluation of the electrocatalytic activity of electrocatalysts with different properties (e.g., chemical composition, nanostructure, etc.).

**Table 1:** The OER performances of the Ir‐based electrocatalysts in acidic condition.

Catalysts	Electrode	Support	Electrolyte	η_10_ [mV]	Tafel slope [mV dec^−1^]	Mass activity [A g^−1^]	TOF [s^−1^]	Stability	PEMWE			Ref.
									Cathode	Overpotential	Stability	
Ir‐Co_3_O_4_	SCE	GCE	0.5 m H_2_SO_4_	236.0	52.60	3343.37	1.67	30 h @10 mA cm^−2^				[72]
Ir_0.08_Co_2.92_O_4_ NWs	Hg/Hg_2_SO_4_	Ti foam	0.5 m H_2_SO_4_	189.5	22.32	1343.10	0.04	100 h @10 mA cm^−2^	Pt/C	1.494V@20 mA cm^−2^	100 h@20 mA cm^−2^	[73]
Co‐IrRu/C‐2	SCE	GCE	0.1 m HClO_4_	248.0	83.00		1.20	3000 cycles				[80]
Ru_0.5_Ir_0.5_	Ag/AgCl	CP	0.5 m H_2_SO_4_	160.0	61.00		10.73	24 h @20 mA cm^−2^	Ru_0.5_Ir_0.5_	1.44V	400 h	[81]
ZnNiCoIrMn	Ag/AgCl	GCE	0.1 m HClO_4_	237.0	46.00	610.80	7.53	100 h @10 mA cm^−2^				[83]
IrFeCoNiCu‐HEA	Ag/AgCl	CP	0.1 m HClO_4_	302.0	58.00	34.67		12 h@10 mA cm^−2^				[84]
IrO_x_/Zr_2_ON_2_	Hg/Hg_2_SO_4_	GCE	0.5 m H_2_SO_4_	255.0	48.00	849.00		5 h@10 mA cm^−2^	Pt/C	1.927 V@2 A cm^−2^	50 h@1 A cm^−2^	[113]
Sr_2_CaIrO_6_	Ag/AgCl	GCE	0.1 m HClO_4_	250.0	33.00	900	0.71	5000 cycles	Pt/C	1.81 V@2 A cm^−2^	450 h@2 A cm^−2^	[155]
Ir‐MnO_2_(160)‐CC	Ag/AgCl	CC	0.5 m H_2_SO_4_	181.0	74.80	343.4		180 h @ 20 mA cm^−2^				[166]
Re_0.1_‐IrO_2_	SCE	CP	0.5 m H_2_SO_4_	255.0	65.60			170 h @ 10 mA cm‐^2^				[148]
BCC‐Cr‐SrIrO_3_	Ag/AgCl	GCE	0.1 m HClO_4_	217.0	54.00	417.6	0.208	40 h @ 10 mA cm^−2^				[153]
4CeO_2_@SrIrO_3_	SCE	GCE	0.5 m H_2_SO_4_	238.0	71.70	249		50 h@10 mA cm‐^2^	Pt/C	1.51 V@10 mA cm^−2^	50 h@10 mA cm^−2^	[89]
IrO_2_/BN	Ag/AgCl	GCE	0.5 m H_2_SO_4_	315.0		1		5 h@10 mA cm^−2^				[138]
H‐γ‐SIO‐1	SCE	GCE	0.1 m HClO_4_	200.0		466		1080 h@10 mA cm^−2^				[156]
Ti‐IrO_x_/Ir			0.5 m H_2_SO_4_	254.0	48.00	338		12 h@100 mA cm^−2^	Pt/C	1.774 V@2 A cm^−2^	220 h@2 A cm^−2^	[99]
Ir‐Sn PSC	Hg/Hg_2_SO_4_	GCE	0.5 m H_2_SO_4_	225.0	64.10	4400	1.06	180 h@30 mA cm^−2^	Pt/C	1.711 V@2 A cm^−2^	260 h@20 mA cm^−2^	[109]
(Ir_0.06_Mn_0.94_)O_1.58_	Ag/AgCl	FTO	0.1 m HClO_4_	260.0	48.00	2043						[103]

**Table 2:** The OER performances of the Ru‐based electrocatalysts in acidic condition.

Catalysts	Electrode	Support	Electrolyte	*η_10_* [mV]	Tafel slope [mV dec^−1^]	Mass activity [A g^−1^]	TOF [s^−1^]	Stability	PEMWE			Ref.
									Cathode	Overpotential	Stability	
Ru/Co–N–C	SCE	CP	0.5 m H_2_SO_4_	232.0			9.20	20 h@10 mA cm^−2^	Ru/Co–N–C	1.49 V@10 mA cm^−2^	330 h@450 mA cm^−2^	[74]
Ni‐RuO_2_	SCE	GCE	0.1 m HClO_4_	214.0	42.6			200 h@10 mA cm^−2^	Pt/C	2.10 V@1.5 A cm^−2^	1000 h@200 mA cm^−2^	[149]
NC@Vo·‐RuO_2_/CNTs‐350	Hg/Hg_2_SO_4_	GCE	0.5 m H_2_SO_4_	170.0	38.9	1738.59		900 h@10 mA cm^−2^	Pt/C	1.45 V@10 mA cm^−2^	1000 h@10 mA cm^−2^	[87]
Co_3_O_4_@CoRuO_x_	Ag/AgCl	CC	0.5 m H_2_SO_4_	161.0	47.9	2069		40 h@10 mA cm^−2^				[100]
RuCuO_x_ NS	Hg_2_/HgCl_2_	CP	0.5 m H_2_SO_4_	175.0	43.6	650	0.39	50 h@10 mA cm^−2^				[101]
SnRuOx	Hg/Hg_2_SO_4_	Gold	0.5 m H_2_SO_4_	194.0	38.2	2360	0.63	250 h@100 mA cm^−2^	Pt/C	1.735 V@3 A cm^−2^	1300 h@1 A cm^−2^	[152]
Si‐RuOx@C	Ag/AgCl	GCE	0.5 m H_2_SO_4_	220.0	53.0	400.2		100 h@10 mA cm^−2^				[150]
Ru@V‐RuO_2_/C HMS	Ag/AgCl	GCE	0.5 m H_2_SO_4_	176.0	45.6			5000 cycles	Ru@V‐RuO_2_/C HMS	1.467 V@10 mA cm^−2^	25 h@10 mA cm^−2^	[110]
Re_0.06_Ru_0.94_O_2_	Ag/AgCl	GCE	0.1 m HClO_4_	190.0	45.5	500	0.17	200 h@10 mA cm^−2^				[147]
Y_1.75_Co_0.25_Ru_2_O_7−δ_	Ag/AgCl	GCE	0.5 m H_2_SO_4_	275.0	61.0			2000 cycles				[91]
Ru_5_W_1_O_x_	Hg/Hg_2_SO_4_	GCE	0.5 m H_2_SO_4_	227.0	42.0	547	0.163	100 h @100 mA cm^−2^				[94]
Nb_0.1_Ru_0.9_O_2_	Ag/AgCl	CP	0.5 m H_2_SO_4_	204.0	47.9	150.5		360 h@200 mA cm^−2^	Pt/C	1.69 V@1 A cm^−2^	100 h@300 mA cm^−2^	[144]
Rh‐RuO_2_/G	Ag/AgCl	GCE	0.5 m H_2_SO_4_	161.0	45.8		1.74	500 h@100 mA cm^−2^	Pt/C	1.42 V@10 mA cm^−2^	160 h@10 mA cm^−2^	[159]
Mo‐YRO	SCE	GCE	0.1 m HClO_4_	240.0	40.5	580		30 h@10 mA cm^−2^	Pt/C	1.48 V@50 mA cm^−2^	50 h@50 mA cm^−2^	[108]
Nd_0.1_RuO_x_/CC	Hg/Hg_2_SO_4_	CC	0.5 m H_2_SO_4_	211.0	50.0			25 h@10 mA cm^−2^	Pt/C	1.595 V@50 mA cm^−2^	50 h@10 mA cm^−2^	[145]
RuNiO_x_	Ag/AgCl	Ti foam	0.05 m H_2_SO_4_	217.0	56.0			100 h@10 mA cm^−2^				[49]
H/d‐MnOx/RuO_2_	Hg/Hg_2_SO_4_	GCE	0.5 m H_2_SO_4_	178.0	43.8	500		40 h@10 mA cm^−2^	Pt/C	1.46 V@10 mA cm^−2^	25 h@10 mA cm^−2^	[41]
RuIrAl	Ag/AgCl	GCE	0.5 m H_2_SO_4_	178.0	49.2	120	0.0351	300 h@100 mA cm^−2^	Pt/C	1.536 V@1 A cm^−2^	60 h@1 A cm^−2^	[98]

### 5.5 Expansion of Application Possibilities

To apply these electrocatalysts in practical electrolyzers, in addition to the issues, several aspects need to be considered. First, it is necessary to develop simple and scalable routes for catalyst synthesis to achieve large‐scale production as per demand. Second, binder‐free electrocatalysts should be constructed into hierarchical structures to eliminate the hindrance of binders on charge transfer and stability, thereby enhancing the density of available active sites. Finally, integrating electrolysis systems with solar energy and wind energy provides the optimum solution for converting solar and wind energy directly into hydrogen energy, enabling the transformation, storage, and subsequent utilization of renewable energy into clean, high‐energy‐density fuels.

## Conflict of Interest

The authors declare no conflict of interest.
